# Tetra-μ-benzoato-bis­[(6-methyl­quino­line)­copper(II)]

**DOI:** 10.1107/S1600536808016516

**Published:** 2008-06-07

**Authors:** Seung Man Yu, Chi-Ho Park, Pan-Gi Kim, Cheal Kim, Youngmee Kim

**Affiliations:** a Department of Fine Chemistry, and Eco-Product and Materials Education Center, Seoul National University of Technology, Seoul 139-743, Republic of Korea; b National Institute of Animal Science (NIAS), RDA, Suwon 441-350, Republic of Korea; c Department of Forest and Environment Resources, Kyungpook National University, Sangju 742-711, Republic of Korea; d Department of Chemistry and Nano Sciences, Ewha Womans University, Seoul 120-750, Republic of Korea

## Abstract

In the title compound, [Cu_2_(C_7_H_5_O_2_)_4_(C_10_H_9_N)_2_], the paddle-wheel-type dinuclear complex is constructed by four bridging benzoate groups and two terminal 6-methyl­quinoline ligands. The asymmetric unit contains one-half of the whole mol­ecule, and there is an inversion center at the mid-point of the Cu⋯Cu bond. The octa­hedral coordination of each Cu atom, with four O atoms in the equatorial plane, is completed by the N atom of the 6-methyl­quinoline mol­ecule [Cu—N = 2.212 (2) Å] and by another Cu atom [Cu⋯Cu = 2.6939 (13) Å]. The Cu atom lies 0.234 Å out of the plane of the four O atoms. The molecular packing is stabilized by one intramolecular C—H⋯O as well as C—H⋯π and π–π interactions.

## Related literature

For related literature, see: Batten & Robson (1998 ▶); Chun *et al.* (2005 ▶); Cotton & Walton (1993 ▶); Janiak (2003 ▶); Lee *et al.* (2008 ▶); Mines *et al.* (2002 ▶); Pichon *et al.* (2007 ▶); Yoo *et al.* (2003 ▶).
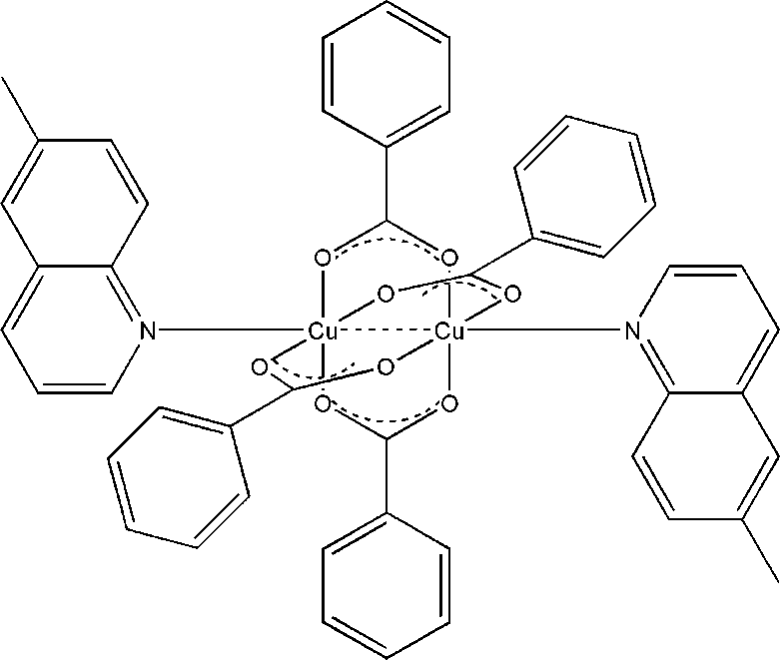


## Experimental

### 

#### Crystal data

[Cu_2_(C_7_H_5_O_2_)_4_(C_10_H_9_N)_2_]
*M* *_r_* = 897.88
Triclinic, 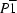

*a* = 10.420 (7) Å
*b* = 10.590 (7) Å
*c* = 10.751 (6) Å
α = 70.399 (11)°
β = 64.234 (10)°
γ = 81.107 (10)°
*V* = 1006.5 (11) Å^3^
*Z* = 1
Mo *K*α radiation
μ = 1.12 mm^−1^
*T* = 288 (2) K
0.10 × 0.08 × 0.08 mm

#### Data collection

Bruker SMART CCD area-detector diffractometer
Absorption correction: multi-scan (*SADABS*: Bruker, 1997 ▶) *T*_min_ = 0.898, *T*_max_ = 0.915
5579 measured reflections
3848 independent reflections
3001 reflections with *I* > 2σ(*I*)
*R*_int_ = 0.021

#### Refinement

*R*[*F* ^2^ > 2σ(*F* ^2^)] = 0.041
*wR*(*F* ^2^) = 0.100
*S* = 1.04
3848 reflections
272 parameters
H-atom parameters constrained
Δρ_max_ = 0.31 e Å^−3^
Δρ_min_ = −0.33 e Å^−3^

### 

Data collection: *SMART* (Bruker, 1997 ▶); cell refinement: *SAINT* (Bruker, 1997 ▶); data reduction: *SAINT*; program(s) used to solve structure: *SHELXS97* (Sheldrick, 2008 ▶); program(s) used to refine structure: *SHELXL97* (Sheldrick, 2008 ▶); molecular graphics: *SHELXTL* (Sheldrick, 2008 ▶); software used to prepare material for publication: *SHELXTL*.

## Supplementary Material

Crystal structure: contains datablocks I, New_Global_Publ_Block. DOI: 10.1107/S1600536808016516/bx2146sup1.cif

Structure factors: contains datablocks I. DOI: 10.1107/S1600536808016516/bx2146Isup2.hkl

Additional supplementary materials:  crystallographic information; 3D view; checkCIF report

## Figures and Tables

**Table 1:** Hydrogen-bond geometry (Å, °) *Cg*1 is the centroid of the C22–C27 ring.

*D*—H⋯*A*	*D*—H	H⋯*A*	*D*⋯*A*	*D*—H⋯*A*
C1—H1⋯O11	0.93	2.50	3.047 (4)	118
C2—H2⋯*Cg*1^i^	0.93	2.82	3.734 (3)	168

Symmetry code: (i) 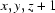.

**Table 2:** π–π interactions (Å, °) *Cg*2 is the centroid of ring C22–C27. The offset is defined as the distance between *CgI* and the perpendicular projection of *CgJ* on ring *I*.

*CgI*	*CgJ*	*CgI*⋯*CgJ*	Dihedral angle	Interplanar distance	Offset
*Cg*2	*Cg*2i	3.967 (4)		3.39	2.06

Symmetry code: (i) 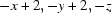.

**Fig. 1.**
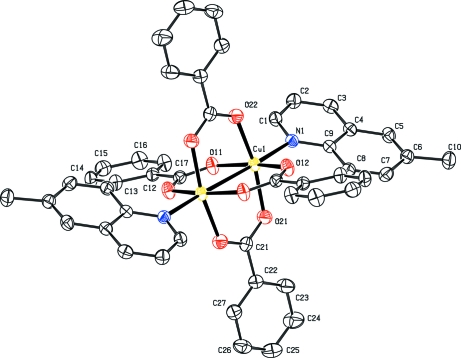
The structure of the title compound showing the atom-labeling scheme. Displacement ellipsoids are shown at the 30% probability level.

## supplementary crystallographic information

### Comment

Coordination polymers comprised of metal ions and bridging ligands represent one of the most active areas of material science and chemical research due to their potential applications as functional materials ranging from catalysis, gas absorption, molecular recognition, optics, and so on (Batten & Robson, 1998; Chun *et al.*, 2005; Mines *et al.*, 2002; Janiak, 2003; Yoo *et al.*, 2003). The continuing interest in this area is also due to their intriguing variety of architectures and topologies through the variation of building blocks and reaction conditions. The dinuclear metal carboxylates, *M*_2_(O_2_CR)_4_, are one of the important building blocks for the study of structures of coordination polymers (Cotton & Walton, 1993) and copper(II) carboxylates among them are often used as building blocks to form a pillard-grid MOF with large pores (Pichon *et al.*, 2007). We have also used copper(II) benzoate as a building block and reported the structure of copper(II) benzoate with quinoxaline (Lee, *et al.*, 2008). In this work, we have employed 6-methylquinoline to investigate the substituent effect of an organic ligand on the structure of copper-benzoate containing coordination complexes.We report here on the structure of new copper(II) benzoate with 6-methylquinoline.

Asymmetric unit contains half of whole molecule, and there is an inversion center in the middle of Cu—Cu bond. Symmetric operation (-*x* + 1,-*y* + 2,-*z* + 1) produces a paddle-wheel type dinuclear copper-benzoate complex (Fig. 1). The paddle-wheel type dinuclear complex is constructed by four bridging benzoate groups and two terminal 6-methylquinoline ligands. The octahedral coordination of each Cu atom, with four oxygen atoms in the equatorial plane, is completed by nitrogen atom of 6-methylquinoline molecule (Cu—N 2.212 (2) Å) and by another copper atom (Cu···Cu 2.6939 (13) Å). The copper atom is 0.234 Å out of the plane of the four oxygen atoms.In the crystal structure the molecular packing is stabilized by one intramolecular C—H···O as well as C—H···π and π ···π interactions, Table, 1 and 2.

### Experimental

19.0 mg (0.1 mmol) of Cu(NO_3_)_2_^.^2.5H_2_O and 28.0 mg (0.2 mmol) of C_6_H_5_COONH_4_ were dissolved in 4 ml me thanol and carefully layered by 4 ml acetone solution of 6-methylquinoline ligand (29.0 mg, 0.2 mmol). Suitable crystals of the title compound for X-ray analysis were obtained in a few weeks.

### Refinement

(type here to add refinement details)

### Crystal data

**Table:**

[Cu_2_(C_7_H_5_O_2_)_4_(C_10_H_9_N)_2_]	*Z* = 1
*M**_r_* = 897.88	*F*_000_ = 462
Triclinic, *P*1	*D*_x_ = 1.481 Mg m^−^^3^
*a* = 10.420 (7) Å	Mo *K*α radiation λ = 0.71073 Å
*b* = 10.590 (7) Å	Cell parameters from 1441 reflections
*c* = 10.751 (6) Å	θ = 2.4–19.8º
α = 70.399 (11)º	µ = 1.12 mm^−^^1^
β = 64.234 (10)º	*T* = 288 (2) K
γ = 81.107 (10)º	Block, blue
*V* = 1006.5 (11) Å^3^	0.10 × 0.08 × 0.08 mm

### Data collection

**Table:**

Bruker SMART CCD area-detector diffractometer	3848 independent reflections
Radiation source: fine-focus sealed tube	3001 reflections with *I* > 2σ(*I*)
Monochromator: graphite	*R*_int_ = 0.021
*T* = 288(2) K	θ_max_ = 26.0º
phi and ω scans	θ_min_ = 2.0º
Absorption correction: multi-scan(SADABS: Bruker, 1997)	*h* = −12→12
*T*_min_ = 0.898, *T*_max_ = 0.915	*k* = −13→10
5579 measured reflections	*l* = −13→9

### Refinement

**Table:**

Refinement on *F*^2^	Secondary atom site location: difference Fourier map
Least-squares matrix: full	Hydrogen site location: inferred from neighbouring sites
*R*[*F*^2^ > 2σ(*F*^2^)] = 0.041	H-atom parameters constrained
*wR*(*F*^2^) = 0.100	*w* = 1/[σ^2^(*F*_o_^2^) + (0.0506*P*)^2^] where *P* = (*F*_o_^2^ + 2*F*_c_^2^)/3
*S* = 1.05	(Δ/σ)_max_ < 0.001
3848 reflections	Δρ_max_ = 0.31 e Å^−^^3^
272 parameters	Δρ_min_ = −0.33 e Å^−^^3^
Primary atom site location: structure-invariant direct methods	Extinction correction: none

### Special details

**Table:**

Geometry. All e.s.d.'s (except the e.s.d. in the dihedral angle between two l.s. planes) are estimated using the full covariance matrix. The cell e.s.d.'s are taken into account individually in the estimation of e.s.d.'s in distances, angles and torsion angles; correlations between e.s.d.'s in cell parameters are only used when they are defined by crystal symmetry. An approximate (isotropic) treatment of cell e.s.d.'s is used for estimating e.s.d.'s involving l.s. planes.
Refinement. Refinement of *F*^2^ against ALL reflections. The weighted *R*-factor *wR* and goodness of fit *S* are based on *F*^2^, conventional *R*-factors *R* are based on *F*, with *F* set to zero for negative *F*^2^. The threshold expression of *F*^2^ > σ(*F*^2^) is used only for calculating *R*-factors(gt) *etc*. and is not relevant to the choice of reflections for refinement. *R*-factors based on *F*^2^ are statistically about twice as large as those based on *F*, and *R*- factors based on ALL data will be even larger.

### Fractional atomic coordinates and isotropic or equivalent isotropic displacement parameters (Å^2^)

**Table:**

	*x*	*y*	*z*	*U*_iso_*/*U*_eq_	
Cu1	0.52984 (3)	0.89016 (3)	0.59215 (4)	0.03698 (14)	
N1	0.6052 (2)	0.7261 (2)	0.7373 (2)	0.0381 (6)	
C1	0.6560 (3)	0.7658 (3)	0.8104 (3)	0.0443 (7)	
H1	0.6566	0.8574	0.7958	0.053*	
C2	0.7091 (3)	0.6802 (3)	0.9084 (3)	0.0468 (8)	
H2	0.7434	0.7144	0.9572	0.056*	
C3	0.7097 (3)	0.5470 (3)	0.9310 (3)	0.0442 (7)	
H3	0.7447	0.4885	0.9959	0.053*	
C4	0.6576 (3)	0.4967 (3)	0.8567 (3)	0.0378 (7)	
C5	0.6493 (3)	0.3595 (3)	0.8781 (3)	0.0449 (7)	
H5	0.6834	0.2980	0.9421	0.054*	
C6	0.5935 (3)	0.3131 (3)	0.8091 (3)	0.0442 (7)	
C7	0.5463 (3)	0.4077 (3)	0.7091 (3)	0.0476 (8)	
H7	0.5104	0.3775	0.6587	0.057*	
C8	0.5519 (3)	0.5417 (3)	0.6840 (3)	0.0433 (7)	
H8	0.5203	0.6016	0.6169	0.052*	
C9	0.6053 (3)	0.5905 (3)	0.7591 (3)	0.0366 (6)	
C10	0.5756 (4)	0.1656 (3)	0.8414 (4)	0.0595 (9)	
H10A	0.6670	0.1251	0.8005	0.089*	
H10B	0.5150	0.1533	0.8001	0.089*	
H10C	0.5332	0.1243	0.9444	0.089*	
O11	0.6763 (2)	1.01228 (19)	0.5543 (2)	0.0489 (5)	
O12	0.3714 (2)	0.8060 (2)	0.5994 (2)	0.0487 (5)	
C11	0.6982 (3)	1.1320 (3)	0.4734 (3)	0.0391 (7)	
C12	0.8146 (3)	1.2041 (3)	0.4667 (3)	0.0383 (7)	
C13	0.8366 (3)	1.3391 (3)	0.3937 (3)	0.0469 (8)	
H13	0.7792	1.3862	0.3465	0.056*	
C14	0.9423 (3)	1.4042 (3)	0.3903 (3)	0.0530 (8)	
H14	0.9554	1.4954	0.3417	0.064*	
C15	1.0290 (3)	1.3360 (3)	0.4580 (3)	0.0549 (9)	
H15	1.1012	1.3805	0.4549	0.066*	
C16	1.0084 (3)	1.2030 (3)	0.5295 (4)	0.0576 (9)	
H16	1.0677	1.1561	0.5744	0.069*	
C17	0.9012 (3)	1.1371 (3)	0.5361 (3)	0.0516 (8)	
H17	0.8867	1.0465	0.5877	0.062*	
O21	0.6535 (2)	0.8478 (2)	0.4116 (2)	0.0510 (6)	
O22	0.3913 (2)	0.9690 (2)	0.7424 (2)	0.0487 (5)	
C21	0.6732 (3)	0.9229 (3)	0.2858 (3)	0.0391 (7)	
C22	0.7844 (3)	0.8779 (3)	0.1630 (3)	0.0409 (7)	
C23	0.8502 (4)	0.7562 (4)	0.1888 (4)	0.0623 (10)	
H23	0.8224	0.6991	0.2831	0.075*	
C24	0.9574 (4)	0.7171 (4)	0.0763 (4)	0.0760 (12)	
H24	1.0019	0.6341	0.0950	0.091*	
C25	0.9985 (4)	0.8004 (4)	−0.0633 (4)	0.0680 (10)	
H25	1.0712	0.7743	−0.1391	0.082*	
C26	0.9323 (4)	0.9216 (4)	−0.0900 (4)	0.0617 (10)	
H26	0.9594	0.9780	−0.1846	0.074*	
C27	0.8256 (3)	0.9609 (3)	0.0223 (3)	0.0502 (8)	
H27	0.7809	1.0438	0.0032	0.060*	

### Atomic displacement parameters (Å^2^)

**Table:**

	*U*^11^	*U*^22^	*U*^33^	*U*^12^	*U*^13^	*U*^23^
Cu1	0.0383 (2)	0.0324 (2)	0.0399 (2)	0.00153 (14)	−0.01945 (16)	−0.00685 (15)
N1	0.0415 (14)	0.0358 (14)	0.0372 (13)	0.0001 (10)	−0.0181 (11)	−0.0090 (10)
C1	0.0492 (18)	0.0365 (17)	0.0453 (18)	−0.0022 (13)	−0.0206 (15)	−0.0078 (13)
C2	0.0510 (19)	0.048 (2)	0.0507 (19)	−0.0009 (14)	−0.0295 (16)	−0.0147 (15)
C3	0.0407 (17)	0.0497 (19)	0.0418 (17)	0.0048 (14)	−0.0221 (14)	−0.0085 (14)
C4	0.0337 (16)	0.0377 (16)	0.0379 (16)	0.0021 (12)	−0.0144 (13)	−0.0079 (13)
C5	0.0423 (17)	0.0361 (17)	0.0494 (19)	0.0059 (13)	−0.0197 (15)	−0.0062 (14)
C6	0.0448 (18)	0.0392 (17)	0.0448 (18)	0.0036 (13)	−0.0168 (15)	−0.0120 (14)
C7	0.0540 (19)	0.0469 (19)	0.0497 (19)	0.0033 (15)	−0.0251 (16)	−0.0206 (15)
C8	0.0528 (19)	0.0393 (17)	0.0401 (17)	−0.0006 (14)	−0.0237 (15)	−0.0085 (13)
C9	0.0367 (16)	0.0363 (16)	0.0331 (15)	−0.0026 (12)	−0.0133 (13)	−0.0068 (12)
C10	0.073 (2)	0.0393 (19)	0.070 (2)	0.0016 (16)	−0.034 (2)	−0.0151 (16)
O11	0.0497 (13)	0.0358 (12)	0.0616 (14)	−0.0046 (9)	−0.0312 (11)	−0.0022 (10)
O12	0.0491 (13)	0.0398 (12)	0.0585 (13)	−0.0039 (10)	−0.0317 (11)	−0.0017 (10)
C11	0.0379 (16)	0.0363 (17)	0.0411 (17)	0.0006 (13)	−0.0132 (14)	−0.0137 (13)
C12	0.0350 (16)	0.0367 (16)	0.0413 (17)	0.0002 (12)	−0.0135 (13)	−0.0128 (13)
C13	0.0485 (19)	0.0387 (18)	0.0525 (19)	−0.0006 (14)	−0.0221 (16)	−0.0104 (14)
C14	0.058 (2)	0.0367 (18)	0.058 (2)	−0.0102 (15)	−0.0184 (17)	−0.0102 (15)
C15	0.0415 (19)	0.066 (2)	0.058 (2)	−0.0121 (16)	−0.0134 (16)	−0.0248 (18)
C16	0.048 (2)	0.060 (2)	0.069 (2)	−0.0029 (16)	−0.0319 (18)	−0.0124 (18)
C17	0.0471 (19)	0.0436 (19)	0.061 (2)	−0.0037 (15)	−0.0251 (17)	−0.0070 (15)
O21	0.0572 (14)	0.0438 (13)	0.0428 (13)	0.0113 (10)	−0.0182 (11)	−0.0104 (10)
O22	0.0509 (13)	0.0469 (13)	0.0484 (12)	0.0108 (10)	−0.0229 (10)	−0.0161 (10)
C21	0.0363 (16)	0.0394 (17)	0.0476 (19)	−0.0013 (13)	−0.0212 (14)	−0.0145 (14)
C22	0.0366 (16)	0.0488 (19)	0.0424 (18)	0.0003 (13)	−0.0196 (14)	−0.0158 (14)
C23	0.064 (2)	0.067 (2)	0.0436 (19)	0.0204 (18)	−0.0191 (18)	−0.0152 (17)
C24	0.070 (3)	0.082 (3)	0.068 (3)	0.037 (2)	−0.027 (2)	−0.032 (2)
C25	0.055 (2)	0.094 (3)	0.055 (2)	0.006 (2)	−0.0153 (19)	−0.036 (2)
C26	0.061 (2)	0.084 (3)	0.0413 (19)	−0.017 (2)	−0.0191 (18)	−0.0156 (18)
C27	0.053 (2)	0.053 (2)	0.050 (2)	−0.0023 (15)	−0.0265 (17)	−0.0140 (16)

### Geometric parameters (Å, °)

**Table:**

Cu1—O12	1.955 (2)	C11—O12^i^	1.254 (3)
Cu1—O21	1.964 (2)	C11—C12	1.495 (4)
Cu1—O11	1.971 (2)	C12—C13	1.380 (4)
Cu1—O22	1.974 (2)	C12—C17	1.381 (4)
Cu1—N1	2.212 (2)	C13—C14	1.367 (4)
Cu1—Cu1^i^	2.6939 (13)	C13—H13	0.9300
N1—C1	1.314 (4)	C14—C15	1.373 (4)
N1—C9	1.375 (4)	C14—H14	0.9300
C1—C2	1.396 (4)	C15—C16	1.359 (4)
C1—H1	0.9300	C15—H15	0.9300
C2—C3	1.347 (4)	C16—C17	1.370 (4)
C2—H2	0.9300	C16—H16	0.9300
C3—C4	1.404 (4)	C17—H17	0.9300
C3—H3	0.9300	O21—C21	1.261 (3)
C4—C5	1.403 (4)	O22—C21^i^	1.250 (3)
C4—C9	1.419 (4)	C21—O22^i^	1.250 (3)
C5—C6	1.355 (4)	C21—C22	1.495 (4)
C5—H5	0.9300	C22—C23	1.364 (4)
C6—C7	1.410 (4)	C22—C27	1.380 (4)
C6—C10	1.505 (4)	C23—C24	1.379 (5)
C7—C8	1.358 (4)	C23—H23	0.9300
C7—H7	0.9300	C24—C25	1.372 (5)
C8—C9	1.410 (4)	C24—H24	0.9300
C8—H8	0.9300	C25—C26	1.363 (5)
C10—H10A	0.9600	C25—H25	0.9300
C10—H10B	0.9600	C26—C27	1.377 (5)
C10—H10C	0.9600	C26—H26	0.9300
O11—C11	1.262 (3)	C27—H27	0.9300
O12—C11^i^	1.254 (3)		
			
O12—Cu1—O21	89.07 (10)	H10A—C10—H10C	109.5
O12—Cu1—O11	166.38 (8)	H10B—C10—H10C	109.5
O21—Cu1—O11	89.52 (10)	C11—O11—Cu1	127.61 (19)
O12—Cu1—O22	88.79 (10)	C11^i^—O12—Cu1	121.26 (19)
O21—Cu1—O22	166.32 (8)	O12^i^—C11—O11	124.7 (3)
O11—Cu1—O22	89.39 (10)	O12^i^—C11—C12	118.4 (3)
O12—Cu1—N1	101.96 (9)	O11—C11—C12	116.9 (3)
O21—Cu1—N1	97.02 (9)	C13—C12—C17	118.5 (3)
O11—Cu1—N1	91.66 (9)	C13—C12—C11	121.1 (3)
O22—Cu1—N1	96.64 (10)	C17—C12—C11	120.4 (3)
O12—Cu1—Cu1^i^	86.24 (7)	C14—C13—C12	120.4 (3)
O21—Cu1—Cu1^i^	82.33 (7)	C14—C13—H13	119.8
O11—Cu1—Cu1^i^	80.14 (7)	C12—C13—H13	119.8
O22—Cu1—Cu1^i^	84.05 (7)	C13—C14—C15	120.6 (3)
N1—Cu1—Cu1^i^	171.77 (6)	C13—C14—H14	119.7
C1—N1—C9	117.2 (2)	C15—C14—H14	119.7
C1—N1—Cu1	114.60 (19)	C16—C15—C14	119.3 (3)
C9—N1—Cu1	128.17 (19)	C16—C15—H15	120.4
N1—C1—C2	124.6 (3)	C14—C15—H15	120.4
N1—C1—H1	117.7	C15—C16—C17	120.7 (3)
C2—C1—H1	117.7	C15—C16—H16	119.6
C3—C2—C1	118.8 (3)	C17—C16—H16	119.6
C3—C2—H2	120.6	C16—C17—C12	120.5 (3)
C1—C2—H2	120.6	C16—C17—H17	119.8
C2—C3—C4	120.0 (3)	C12—C17—H17	119.8
C2—C3—H3	120.0	C21—O21—Cu1	125.3 (2)
C4—C3—H3	120.0	C21^i^—O22—Cu1	123.10 (19)
C5—C4—C3	123.6 (3)	O22^i^—C21—O21	125.0 (3)
C5—C4—C9	118.7 (3)	O22^i^—C21—C22	118.6 (3)
C3—C4—C9	117.7 (3)	O21—C21—C22	116.4 (3)
C6—C5—C4	122.6 (3)	C23—C22—C27	119.1 (3)
C6—C5—H5	118.7	C23—C22—C21	120.4 (3)
C4—C5—H5	118.7	C27—C22—C21	120.5 (3)
C5—C6—C7	118.0 (3)	C22—C23—C24	120.6 (3)
C5—C6—C10	121.9 (3)	C22—C23—H23	119.7
C7—C6—C10	120.1 (3)	C24—C23—H23	119.7
C8—C7—C6	121.9 (3)	C25—C24—C23	120.1 (4)
C8—C7—H7	119.0	C25—C24—H24	119.9
C6—C7—H7	119.0	C23—C24—H24	119.9
C7—C8—C9	120.3 (3)	C26—C25—C24	119.6 (3)
C7—C8—H8	119.8	C26—C25—H25	120.2
C9—C8—H8	119.8	C24—C25—H25	120.2
N1—C9—C8	119.9 (2)	C25—C26—C27	120.3 (3)
N1—C9—C4	121.7 (3)	C25—C26—H26	119.9
C8—C9—C4	118.4 (3)	C27—C26—H26	119.9
C6—C10—H10A	109.5	C26—C27—C22	120.3 (3)
C6—C10—H10B	109.5	C26—C27—H27	119.8
H10A—C10—H10B	109.5	C22—C27—H27	119.8
C6—C10—H10C	109.5		
			
O12—Cu1—N1—C1	−147.0 (2)	Cu1^i^—Cu1—O12—C11^i^	1.0 (2)
O21—Cu1—N1—C1	122.5 (2)	Cu1—O11—C11—O12^i^	−0.8 (4)
O11—Cu1—N1—C1	32.8 (2)	Cu1—O11—C11—C12	178.41 (18)
O22—Cu1—N1—C1	−56.8 (2)	O12^i^—C11—C12—C13	6.2 (4)
O12—Cu1—N1—C9	33.2 (2)	O11—C11—C12—C13	−173.0 (3)
O21—Cu1—N1—C9	−57.3 (2)	O12^i^—C11—C12—C17	−175.0 (3)
O11—Cu1—N1—C9	−147.0 (2)	O11—C11—C12—C17	5.7 (4)
O22—Cu1—N1—C9	123.4 (2)	C17—C12—C13—C14	0.2 (5)
C9—N1—C1—C2	−0.3 (4)	C11—C12—C13—C14	179.0 (3)
Cu1—N1—C1—C2	179.9 (2)	C12—C13—C14—C15	0.7 (5)
N1—C1—C2—C3	0.2 (5)	C13—C14—C15—C16	−0.4 (5)
C1—C2—C3—C4	0.0 (5)	C14—C15—C16—C17	−0.9 (5)
C2—C3—C4—C5	−177.4 (3)	C15—C16—C17—C12	1.8 (5)
C2—C3—C4—C9	−0.1 (4)	C13—C12—C17—C16	−1.5 (5)
C3—C4—C5—C6	177.4 (3)	C11—C12—C17—C16	179.7 (3)
C9—C4—C5—C6	0.1 (4)	O12—Cu1—O21—C21	90.1 (2)
C4—C5—C6—C7	2.0 (5)	O11—Cu1—O21—C21	−76.4 (2)
C4—C5—C6—C10	−175.4 (3)	O22—Cu1—O21—C21	9.1 (5)
C5—C6—C7—C8	−1.9 (5)	N1—Cu1—O21—C21	−168.0 (2)
C10—C6—C7—C8	175.6 (3)	Cu1^i^—Cu1—O21—C21	3.8 (2)
C6—C7—C8—C9	−0.3 (5)	O12—Cu1—O22—C21^i^	−85.7 (2)
C1—N1—C9—C8	179.9 (3)	O21—Cu1—O22—C21^i^	−4.6 (5)
Cu1—N1—C9—C8	−0.3 (4)	O11—Cu1—O22—C21^i^	80.8 (2)
C1—N1—C9—C4	0.2 (4)	N1—Cu1—O22—C21^i^	172.4 (2)
Cu1—N1—C9—C4	−179.98 (18)	Cu1^i^—Cu1—O22—C21^i^	0.6 (2)
C7—C8—C9—N1	−177.4 (3)	Cu1—O21—C21—O22^i^	−5.7 (4)
C7—C8—C9—C4	2.3 (4)	Cu1—O21—C21—C22	173.24 (18)
C5—C4—C9—N1	177.5 (2)	O22^i^—C21—C22—C23	−175.4 (3)
C3—C4—C9—N1	0.0 (4)	O21—C21—C22—C23	5.6 (4)
C5—C4—C9—C8	−2.2 (4)	O22^i^—C21—C22—C27	6.9 (4)
C3—C4—C9—C8	−179.7 (3)	O21—C21—C22—C27	−172.1 (3)
O12—Cu1—O11—C11	−1.8 (5)	C27—C22—C23—C24	0.8 (5)
O21—Cu1—O11—C11	82.3 (3)	C21—C22—C23—C24	−177.0 (3)
O22—Cu1—O11—C11	−84.1 (3)	C22—C23—C24—C25	−0.3 (6)
N1—Cu1—O11—C11	179.3 (2)	C23—C24—C25—C26	−0.3 (6)
Cu1^i^—Cu1—O11—C11	0.0 (2)	C24—C25—C26—C27	0.5 (6)
O21—Cu1—O12—C11^i^	−81.4 (2)	C25—C26—C27—C22	−0.1 (5)
O11—Cu1—O12—C11^i^	2.7 (5)	C23—C22—C27—C26	−0.6 (5)
O22—Cu1—O12—C11^i^	85.1 (2)	C21—C22—C27—C26	177.2 (3)
N1—Cu1—O12—C11^i^	−178.4 (2)		

Symmetry codes: (i) −*x*+1, −*y*+2, −*z*+1.

### Hydrogen-bond geometry (Å, °)

**Table:**

*D*—H···*A*	*D*—H	H···*A*	*D*···*A*	*D*—H···*A*
C1—H1···O11	0.93	2.50	3.047 (4)	118
C2—H2···Cg1^ii^	0.93	2.82	3.734 (3)	168

Symmetry codes: (ii) *x*, *y*, *z*+1.

### Table 2 π–π interactions ( Å, ° )

Cg2 is the centroid of ring C22–C27. The offset is defined as the distance between CgI and the perpendicular projection of CgJ on ring I.

**Table:**

CgI	CgJ	CgI···CgJ	Dihedral angle	Interplanar distance	Offset
Cg2	Cg2i	3.967 (4)	0	3.39	2.06

Symmetry code: (i) -x+2,-y+2,-z.
